# A Systematic Analysis of COVID-19 Clinical Trials Registered in the Clinical Trials Registry of India

**DOI:** 10.7759/cureus.57939

**Published:** 2024-04-09

**Authors:** Siddhartha Dutta, Shubha Singhal, Rima Shah

**Affiliations:** 1 Department of Pharmacology, All India Institute of Medical Sciences, Rajkot, Rajkot, IND; 2 Department of Pharmacology, All India institute of Medical Sciences, Rajkot, Rajkot, IND

**Keywords:** sars-cov-2, biologics, drugs, ayurveda, coronavirus, covid-19, ayush, clinical trials, ctri

## Abstract

Background: The Clinical Trials Registry - India (CTRI) database is a registry of various trials conducted in India and this study scrutinized the studies registered for COVID-19 from the database to detect patterns in trial design, appraising the target regions of therapies and comprehending the terrain of research endeavors.

Method: This was a cross-sectional study that analyzed the registered trials for COVID-19 between March 2020 and September 2023. A trial search was conducted on the CTRI database to include all types of studies registered for COVID-19 with keywords like “COVID” and “coronavirus” and studies conducted on conditions other than COVID-19 were excluded. The data regarding study characteristics were noted under various sections in a preformed proforma.

Results: A total of 807 trials were taken for final analysis and there were about 344 prospective and 260 retrospective interventional trials, 35 prospective and 165 retrospective observational studies, and two prospective and one retrospective post-marketing surveillance study. The majority of the studies had duration under 12 months (91%). The maximum number of studies were registered from AYUSH (Ayurveda, Yoga and Naturopathy, Unani, Siddha, and Homeopathy) and allied therapies (n = 283), with about 104 types of interventions, followed by the drug category having 119 trials registered and about 57 types of interventions. Kabasura Kudineer and yoga in the AYUSH category, molnupiravir, colchicine, and favipiravir in the drug category, and tocilizumab and convalescent plasma among biologics were some common interventions used. The majority of trials did not mention the trial phase and declared it as not applicable (54%), whereas 15% were registered as phase 2 and 13% as phase 3. About 54% of the studies were randomized and randomized parallel-group design (20%) was the most common study design. Only 6% of the trials were post-graduate thesis and the majority of the trials (n = 535) denied sharing their individual participant data. Only 0.86% and 0.61% of the trials were terminated and suspended, respectively, denoting proper design and conduct of the trials.

Conclusion: In the CTRI database, the majority of trials were prospective interventional studies, with a predominance of AYUSH therapies and drug interventions. Common interventions included Kabasura Kudineer and yoga in AYUSH, and molnupiravir, colchicine, and favipiravir in drugs. Most studies had durations under 12 months and randomized parallel-group design was the most common study design. The intention to use and promote an indigenous system of medicine looks promising in the absence of any definite therapy. A minute number of registered suspended and terminated trials might be a positive picture of meticulously designed and executed trials even during a pandemic situation in India.

## Introduction

A clinical trial, as defined by the National Institutes of Health (NIH), is a “research study in which one or more human subjects are prospectively assigned to one or more interventions (which may include a placebo or other control) to evaluate the effects of those interventions on health-related biomedical or behavioral outcomes” [1]. Clinical trials always depend on the participation of volunteers and also involve a significant investment of human, physical, and financial resources. Some of the trials may terminate for a variety of reasons failing to meet their intended goals, which puts a burden on financial, ethical, and scientific concerns. There can be scientific reasons for which a trial can be terminated that are justifiable, for example, terminating a trial for some safety or efficacy-related issues may prevent additional patient exposure to inefficacious or unfavorable treatments and further curtail the expenses on resources on futile approaches [2,3]. Premature undue termination of any trial other than scientific ground can be due to improper planning or protocol, which can end up in not meeting the intended objectives [4]. To conduct a clinical trial, it takes a lot of money and resources apart from the involvement of the participants, and if a trial gets prematurely terminated, it is a complete loss of all the valuable resources used in addition to the undue trouble to the patient.

With the start of the pandemic associated with coronavirus disease 2019 (COVID-19) in early 2020, the healthcare system across the world compromised to cope with this unprecedented crisis. Due to lack of treatment, existent drugs were being repurposed and there has been a lot of research on the virus and disease to control and cure the condition [5-8]. Apart from investigating the therapies or vaccines for COVID-19, there was also strict monitoring of the associated adverse events to develop a safe and effective therapy for the unprecedented deadly pandemic [9-11]. In India, all the clinical trials are expected to be registered in the Clinical Trials Registry - India (CTRI) database, which is hosted by the Indian Council of Medical Research's (ICMR) National Institute of Medical Statistics [12]. In a recent change, CTRI has instructed that any trial involving human participants, of any intervention, such as drugs, surgical procedures, preventive measures, lifestyle modifications, devices, educational or behavioral treatment, rehabilitation strategies as well as trials being conducted in the purview of the Department of AYUSH, should be registered in the CTRI before enrollment of the first participant [13]. Therefore, the CTRI database now not only includes interventional trials but also includes observational studies, post-marketing surveillance (PMS) studies as well as bioequivalence/bioavailability (BA/BE) trials [13]. With all the COVID-19 trials registered in a single database, we planned this study to systematically evaluate the CTRI database to assess the types of trials conducted on COVID-19 to get an overall picture of the COVID-19 trials conducted in India, including the complementary and alternative medicine trials, and the information from these would help to identify lacunae in the terminated or suspended studies as well as help to plan and guide future clinical research in India.

This study has been planned with the objective of analyzing the various types of COVID-19 trials registered in the CTRI database to understand the facts like appraising the emphasis regions of treatments, recognizing patterns in trial design, and comprehending the terrain of research endeavors and evaluating the suspended and terminated trials on COVID-19 posted on the CTRI results database to determine reasons for their termination. The CTRI database records were searched from March 2020 to September 2023 to access the registered studies from the website http://ctri.nic.in/Clinicaltrials/advancesearchmain.php.

## Materials and methods

This study adopted a cross-sectional study design, obtaining the data of trials registered on the CTRI website. The CTRI website (www.ctri.nic.in) is administered jointly by the ICMR and the National Institute of Medical Statistics.

Trial selection strategy

**Figure 1:**
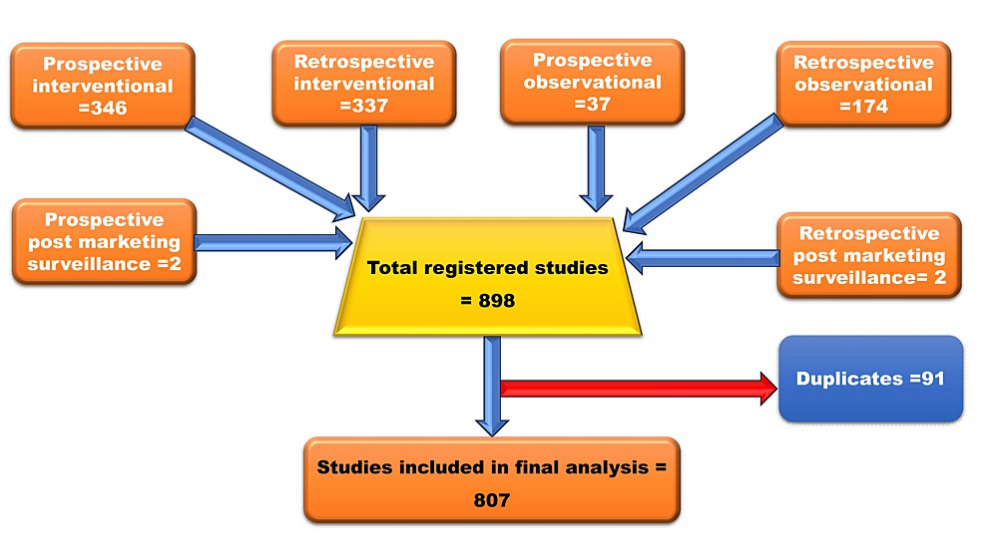
Details of the trials selection strategy

The trials on COVID-19 were identified from the CTRI database (www.ctri.nic.in) and the search was conducted from March 2020 to September 2023. The registered trials were searched in the "trial search" section using keywords such as “COVID” and “coronavirus” to extract all the possible trials registered. For the extraction of terminated trials, the database with filters of recruitment status (Indian) of other (terminated) and terminated trials with the keywords "COVID" and "coronavirus" was searched. Likewise, for the suspended trials, the recruitment status (Indian) for suspended trials with similar keywords was searched. The details of the selection of trials are depicted in Figure 1.

Inclusion and exclusion criteria

Any study conducted on COVID-19 or related to coronavirus was included. We included both prospective and retrospective studies. All types of studies, such as interventional, observational, PMS, and bioavailability and bioequivalence (BA/BE) studies, during the mentioned time frame were included. Studies conducted on conditions other than COVID-19 or coronavirus were excluded.

Data extraction

The search was conducted using the following 11 search elements in the trial search section of the CTRI website with the mentioned search terms: (1) prospective/retrospective trials: both; (2) type of trial: interventional, observational, PMS, and BA/BE; (3) month and year of trial registration: for all the months from March 2020 to September 2023; (4) type of study: ALL; (5) study design: ALL; (6) trial phase: ALL; (7) primary sponsor: ALL; (8) recruitment status (Indian): ALL; (9) state: ALL; (10) district: ALL; (11) keyword: “COVID” or “coronavirus.”

During the initial scrutiny of the registered trials, the duplicate entries with the registry of COVID and coronavirus were removed. Subsequently, the trial protocols were read and the trials were screened for the inclusion criteria, i.e., trials related to or conducted on COVID-19. Further, as the search was conducted through the trial search function, there were many entries where trials were not related to COVID but were picked up in the search because some of the registered trials had used keywords like "COVID" or "coronavirus" anywhere in their protocol registry (for example, in exclusion criteria or related to COVID 19 vaccination). All these non-COVID trials were excluded. On further critical scrutiny, duplicate entries between various types of study designs were also removed to get the final list of studies for analysis. The data were extracted under sections for CTRI number, public title, type of trial, health condition, intervention name, registered on, last modified on, post-graduate thesis, type of study, study design, phase of trial, date of first enrollment (India), date of study completion (India), date of first enrollment (global), date of study completion (global), estimated duration of trial, publication details, brief summary, and individual participant data (IPD) sharing statement for the various COVID-19 registered trials and entered in a preformed proforma in a spreadsheet.

For further analysis of the data, the studies were further divided based on types of studies, types of intervention used like drug, biologics, and stem cells, and AYUSH (Ayurveda, Yoga and Naturopathy, Unani, Siddha, and Homeopathy), intervention name, phases of trials, and study designs. The interventions used were further critically screened, analyzed, and classified to assess the types of interventions used under categories of drugs, biologics, AYUSH, and allied systems.

Ethical approval

This study protocol was approved by the Institutional Ethics Committee of AIIMS Rajkot with certificate reference number AIIMS/IEC/02/2023 (dated: 14th February 2023).

Statistical analysis

Descriptive analyses were used for data analysis, and for categorical variables, the values were expressed as frequencies and percentages, whereas for continuous data, median and interquartile range (IQR) were used.

## Results

The systematic analysis of the database led to the retrieval of 898 studies registered for COVID-19 and coronaviruses, and after removing duplicates, 807 studies were included in the current analysis. The details of the search strategy and included studies are depicted in Figure 1.

**Figure 2:**
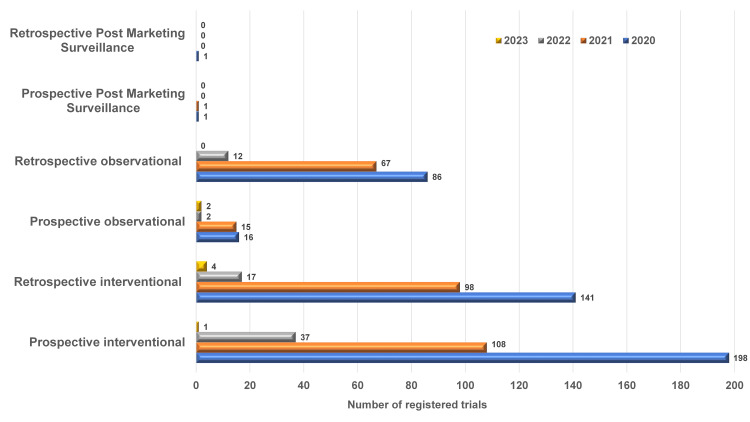
Year-wise details of various types of trials registered for COVID-19 in the CTRI database CTRI: Clinical Trials Registry - India.

Of 807 registered studies, a total of 344 prospective and 260 retrospective interventional trials were registered in the CTRI for COVID-19 during the period of March 2020 to September 2023. Similarly, there were a total of 35 prospective and 165 retrospective observational studies registered during the same timeline. There were two prospective PMS and one retrospective PMS study. Overall, a total of 443 trials were registered in the year 2020, 289 trials in the year 2021, 68 trials in the year 2022, and seven trials in 2023. The detailed breakup of registered trials is depicted in Figure 2.

Duration of studies

The majority of the trials had a planned duration of four to six months (28%), followed by two to four months (24%), zero to two months (17%), and 10-12 months (16%). Almost 69% of the trials had their study duration below six months and about 91% of the trials had their study duration under one year. The mean (SD) time duration for the registered trials was 7.2 (±6.8) months with a median (IQR) of 6 (7.75) months and ranged from zero months to 72 months (Table 1).

**Table 1:** Study duration for the COVID-19 registered trials in CTRI CTRI: Clinical Trials Registry - India.

S. No.	Duration of registered trials	Number of trials
1	0-2 months	140
2	>2 months to 4 months	190
3	>4 months to 6 months	225
4	>6 months to 8 months	16
5	>8 months to 10 months	33
6	>10 months to 12 months	129
7	>12 months to 18 months	31
8	>18 months to 24 months	32
9	>24 months to 36 months	6
10	>36 months to 72 months	4

Types of studies and intervention name

In scrutinizing the types of study section, the studies were classified into various types. Among the broadly divided types, 176 studies were from Ayurveda and allied fields, 129 were drug trials, 92 were cross-sectional studies, 39 were cohort studies, 22 studies were registered for medical devices, 29 studies for nutraceuticals, 18 were diagnostic-related studies, and seven studies were registered with probiotics. The details of all the study types are summarized in Table 2.

**Table 2:** Summary of types of studies registered for COVID-19 trials in CTRI CTRI: Clinical Trials Registry - India; PMS: post-marketing surveillance.

Type of study	Prospective interventional (N = 344), n (%)	Retrospective interventional (N = 260), n (%)	Prospective observational (N = 35), n (%)	Retrospective observational (N = 165), n (%)	Prospective PMS (N = 2), n (%)	Retrospective PMS (N = 1), n (%)
Ayurveda, Ayurveda nutraceutical, Ayurveda others, Ayurveda preventive nutraceutical, Ayurveda Siddha, Ayurveda yoga, Ayurveda yoga & naturopathy, Ayurveda yoga, naturopathy & process of care, drug Ayurveda, drug Ayurveda preventive	101 (29.36)	75 (28.84)	--	--	1 (50)	--
Behavioral	4 (1.16)	2 (0.76)	--	--	--	--
Biological, biological stem cell therapy, drug biological	13 (3.77)	7 (2.69)	--	--	--	--
Case-control	--	--	--	11 (6.66)	--	--
Clinical observational	--	--	--	1 (0.61)	--	--
Cohort	--	--	3 (8.57)	36 (21.81)	--	--
Comparative study	--	--	--	2 (1.21)	--	--
Cross-sectional	--	--	19 (54.28)	73 (44.24)	--	--
Convalescent plasma transfusion, Other specify (convalescent plasma transfusion)	1 (0.29)	1 (0.38)	--	--	--	--
Dentistry, drug dentistry	2 (0.58)	2 (0.76)	--	--	--	--
Descriptive	--	--	1 (2.85)	2 (1.21)	--	--
Diagnostic, diagnostic tool, diagnostic preventive, diagnostic screening, other	5 (1.45)	3 (1.15)	1 (2.85)	3 (1.81)	--	--
Drug	75 (21.80)	53 (20.38)	--	--	1 (50)	
Drug preventive, drug preventive process of care changes, drug process of care changes	4 (1.16)	3 (1.15)	--	--	--	--
Exploratory	--	--	1 (2.85)	--	--	--
Follow-up studies	--	--	4 (11.42)	9 (5.45)	--	--
Homeopathy, homeopathy preventive	17 (4.94)	8 (3.07)	--	--	--	--
Medical device, medical device surgical/anesthesia	11 (3.19)	11 (4.23)	--	--	--	--
Nutraceutical	15 (4.36)	14 (5.38)	--	--	--	--
Non-interventional analytical study	--	--	--	1 (0.60)	--	--
Others, other specify (cosmetics), other specify (hypertonic saline irrigation), other specify (immunological therapy), other specify (inhalation of bioflavonoid complex), other specify (micronutrient supplement), other specify (micronutrient), other specify (monitoring), other specify (non-alcoholic hand sanitizer), other specify (online educational), other specify (oxygen therapy), other specify (psychotherapy), other specify (rehabilitation intervention), other specify (treatment)	17 (4.94)	14 (5.38)	--	--	--	--
Observational research design, non-randomized observational	--	--	1 (2.85)	4 (2.42)	--	--
Physiotherapy	6 (1.74)	3 (1.15)	--	--	--	--
Post-marketing surveillance	--	--	--	--	--	1 (100)
Preventive, preventive process of care changes	3 (0.87)	3 (1.15)	--	--	--	--
Prevalence study	--	--	--	2 (1.21)	--	--
Probiotic, probiotic preventive	3 (0.87)	4 (1.53)	--	--	--	--
Process of care changes	8 (2.32)	5 (1.92)	--	--	--	--
Prospective, prospective observational study of retrospective data, prospective survey, prospective surveillance study, prospective observational	--	--	1 (2.85)	5 (3.03)	--	--
Qualitative study	--	--	--	2 (1.21)	--	--
Questionnaire	--	--	--	2 (1.21)	--	--
Radiation therapy	1 (0.29)	1 (0.38)	--	--	--	--
Registry with ambi-perspective design	--	--	--	1 (0.60)	--	--
Retrospective, retrospective descriptive, single-center, retrospective data collection, retrospective observational, retrospective cohort, retrospective audit, retrospective analysis	--	--	3 (8.57)	11 (6.66)	--	--
Screening	1 (0.29)	1 (0.38)	--	--	--	--
Siddha, Siddha nutraceutical, Siddha preventive, drug Siddha	20 (5.81)	18 (6.92)	--	--	--	--
Stem cell therapy	1 (0.29)	2 (0.76)	--	--	--	--
Surgical/anesthesia	3 (0.87)	4 (1.53)	--	--	--	--
Unani drug, Unani preventive	5 (1.45)	5 (1.92)	--	--	--	--
Vaccine, vaccine Ayurveda, vaccine preventive	11 (3.19)	7 (2.69)	--	--	--	--
Validation	--	--	1 (2.85)	--	--	--
Yoga and naturopathy, yoga and naturopathy (behavioral), yoga, naturopathy and physiotherapy, yoga (others)	17 (4.94)	14 (5.38)	--	--	--	--

A few specific types of trials were further segregated based on broad categories, such as drug, biologics, stem cells, and studies registered as AYUSH along with other allied interventions in these categories, and additionally, the selected study types were analyzed based on the intervention name in each class.

Intervention name - drug

A total of 119 trials were registered in the drug category with about 57 types of interventional drugs that were used. Among them, the majority of the trials registered were molnupiravir (6%), colchicine (6%), favipiravir (5%), ivermectin (5%), dexamethasone (4%), and immunocin α 1.6 mg (4%), respectively (Table 3).

**Table 3:** Summary of all the interventions registered as drug trials in the CTRI database CTRI: Clinical Trials Registry - India; CCB: calcium channel blocker; ARB: angiotensin receptor blocker; MoHFW: Ministry of Health and Family Welfare; BID: twice a day; SOC: standard of care.

S. No.	Intervention name	Number of trials n (%)
1	101-PGC-005	1 (0.84)
2	2-deoxy-D-glucose oral powder	1 (0.84)
3	3mg SNP-ACTH gel sc injection 3 times per week	1 (0.84)
	5mg SNP-ACTH gel sc injection 3 times per week	
	SNP-ACTH (1-39) gel	
4	Acetylsalicylic acid colchicine	1 (0.84)
5	Administration of the drug ivermectin for patients with mild or moderate COVID	2 (1.68)
6	Ampion	2 (1.68)
7	Aspirin, atorvastatin, nicorandil	2 (1.68)
8	Atorvastatin 40 mg	2 (1.68)
9	Aviptadil for injection 500 mcg/vial and standard of care	2 (1.68)
10	Ayurvedic regimen	2 (1.68)
11	Brequinar 50 mg & dipyridamole 75 mg	2 (1.68)
12	BRII-196 and BRII-198 and VIR-7831	2 (1.68)
13	Budesonide 400 mcg	1 (0.84)
14	C21	1 (0.84)
15	CCB and ARB active therapy group	1 (0.84)
	Vitamin D group with age-normalized increase in pulse wave velocity between 364 and 580 cm/sec	
	Vitamin D with CCB and ARB active therapy	
16	CKD-314	2 (1.68)
17	Colchicine	7 (5.88)
	Colchicine 0.5 mg tablets plus standard of care	
	Colchicine, acetylsalicylic acid	
	Colchicine, aspirin, and montelukast	
18	Component A (AP): tablet containing aspirin and promethazine hydrochloride	2 (1.68)
	Component B (MV) multivitamin and multimineral tablet along with standard of care as per MoHFW guidelines	
19	Convalescent plasma	1 (0.84)
20	Dexamethasone	4 (3.36)
21	Eflornithine and SoC	2 (1.68)
22	Essential oil (EO) blend	1 (0.84)
23	Famotidine and celecoxib with standard care	1 (0.84)
24	Favipiravir 200 mg oral tablets	6 (5.04)
	Favipiravir 200 mg tablets and umifenovir capsules: 800 mg BID	
25	Guar gum (Galactomannan) chewable tablet	1 (0.84)
26	Hydroxychloroquine sulfate	2 (1.68)
27	Immunocin α 1.6 mg	4 (3.36)
28	Indomethacin	3 (2.52)
29	Intervention arms 1 and 2	2 (1.68)
30	Intravenous high-dose dexamethasone (20 mg) and tocilizumab 400 mg	2 (1.68)
31	Intravenous silver nanoparticles - AgSept manufactured in addition to standard therapy	2 (1.68)
32	Intravenous vitamin C	2 (1.68)
33	Ivermectin	5 (4.20)
34	Life Viro Treat inhalation	2 (1.68)
35	Losartan	1 (0.84)
36	Molnupiravir	7 (5.88)
37	Montelukast	2 (1.68)
38	Mycophenolate mofetil	2 (1.68)
39	Nafamostat mesilate injection 50 mg/100 mg vial	2 (1.68)
40	Nebulized heparin	2 (1.68)
41	Niclosamide 2000 mg orally plus standard of care	2 (1.68)
42	Normal saline	1 (0.84)
43	Pirfenidone tablet 800 mg	2 (1.68)
44	Purified aqueous extract of *Cocculus hirsutus* (AQCH) tablets	1 (0.84)
45	Recombinant erythropoietin (r-EPO) in addition to standard care	2 (1.68)
46	Remdesivir	1 (0.84)
47	Rivaroxaban	2 (1.68)
48	S-217622	1 (0.84)
49	Shadanga Paneeya Kwath, Vyoshadi Churna tablet, and Vyasthapan Kasay Ghana tablet	2 (1.68)
50	Spironolactone	2 (1.68)
51	Standard of care plus angiotensin receptor blockers (ARB)	2 (1.68)
52	Tab. cefixime 200 mg, Tab. ivermectin 12 mg, Tab. montelukast 10 mg and Syp. Ascoril LS 5 ml	2 (1.68)
53	Tablet sildenafil	1 (0.84)
54	Thymosin α-1-1.6 mg injection along with SOC	2 (1.68)
55	Topical nasal 0.03% chloroquine eye drops	1 (0.84)
56	Topotecan dexamethasone remdesivir	4 (3.36)
57	Vitamin D group with age-normalized increase in pulse wave velocity between 364 and 580 cm/sec	1 (0.84)
	CCB and ARB active therapy group with age-normalized increase in pulse wave velocity	
	Vitamin D with CCB and ARB active therapy group with age-normalized increase in pulse wave velocity	

Intervention name - biologics and stem cells

**Figure 3:**
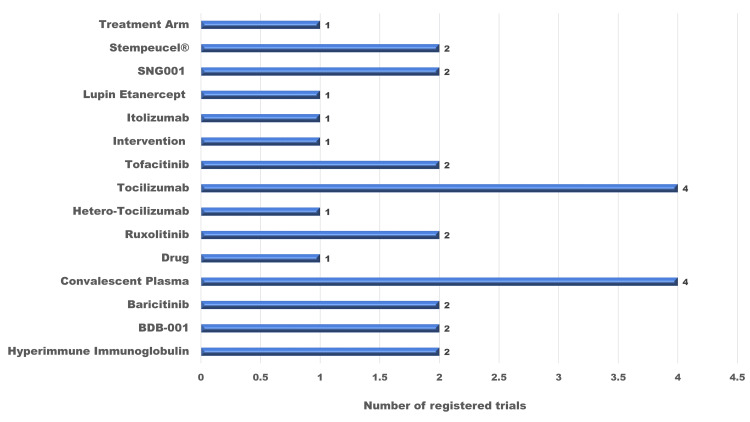
Summary of biologics and stem cell interventions used in trials for COVID-19 registered in the CTRI CTRI: Clinical Trials Registry - India.

A total of 28 trials were registered in the biologics and stem cell therapy section, which included about 15 molecules as interventional agents. The commonest agents that were used under this section were tocilizumab (18%) and convalescent plasma (14%). Other molecules investigated were anti-SARS CoV-2 hyperimmune intravenous immunoglobulin, baricitinib, ruxolitinib, tofacitinib, SNG001, Stempeucel®, and BDB-001 (Figure 3).

Intervention name - AYUSH and associated therapies

On analyzing the trials registered for AYUSH, which included all trials of Ayurveda, the maximum number of interventions were used in this category. A total of 283 trials were registered in this category, and on further subgroup analysis, it was found that about 110 types of AYUSH interventions were tried in COVID-19. A maximum number of trials were registered for Kabasura Kudineer and other associated molecules (5.6%), followed by yoga and allied therapies (5.5%), AYUSH and combined therapies (n = 3.9%), homeopathic medicines (3.2%), Chyawanprash (2.5%), and ashwagandha (1.77%). The details of the AYUSH interventions are mentioned in the Appendix.

Phases of trials

**Figure 4:**
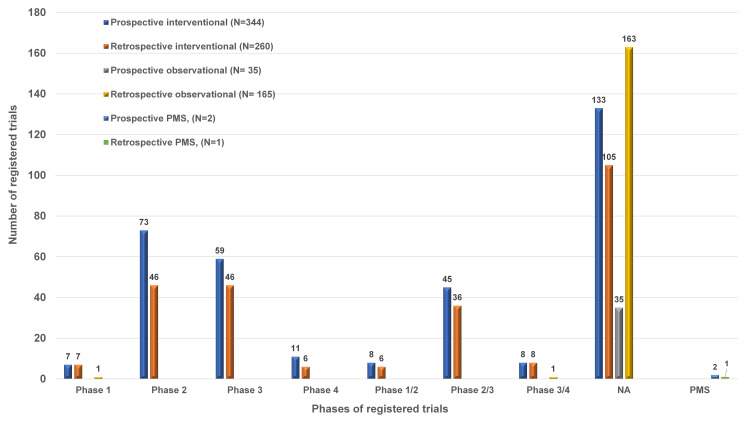
Phase-wise distribution of COVID-19 trials registered in the CTRI CTRI: Clinical Trials Registry - India; PMS: post-marketing surveillance.

On determining the details of the phases of trials, 15 were registered as phase 1, and with phase 2, there were 119 trials registered. A total of 105 phase 3 trials were registered and 17 trials were registered as phase 4. There were three PMS studies. There were about 14 studies registered as phase 1/2, 81 studies as phase 2/3, 17 studies as phase 3/4, and about 436 registered studies had their phase of study as N/A (not applicable). The summary of the phases of trials in COVID-19 studies is depicted in Figure 4.

Study designs

On analyzing the study designs of the registered trials, the majority of them were randomized studies (n = 434, 54%). Among the randomized studies, 162 studies were reported as randomized parallel-group trials, 115 were randomized, parallel-group, active control trials, 121 were randomized, parallel-group, placebo-controlled trials, eight were cluster randomized trials, 19 were randomized multi-arm trials, four were randomized factorial trials, and five were randomized crossover trials. Likewise, 116 research were single-arm studies and 230 of them mentioned their study design as "others" (Table 4).

**Table 4:** Study characteristics of the COVID-19 trials registered in the CTRI database CTRI: Clinical Trials Registry - India.

Study characteristics		Prospective interventional (N = 3 44), n (%)	Retrospective interventional (N = 260), n (%)	Prospective observational (N = 35), n (%)	Retrospective observational (N = 165), n (%)	Prospective PMS (N = 2), n (%)	Retrospective PMS (N = 1), n (%)
Study design	Randomized factorial trial	2 (0.58)	2 (0.76)	--	--	--	--
	Randomized crossover trial	2 (0.58)	3 (1.15)	--	--	--	--
	Randomized parallel-group trial	91 (26.45)	70 (26.92)	--	--	1 (50)	--
	Randomized parallel-group, active control	65 (18.89)	50 (19.23)	--	--	--	--
	Randomized parallel-group, multiple-arm trial	10 (2.90)	9 (3.46)	--	--	--	--
	Randomized parallel-group, placebo-controlled trial	68 (19.76)	53 (20.38)	--	--	--	--
	Cluster randomized trial	5 (1.45)	2 (0.769)	--	1 (0.60)	--	--
	Non-randomized active control trial	8 (2.32)	5 (1.92)	--	--	--	--
	Non-randomized multiple-arm trial	4 (1.16)	3 (1.15)	1 (2.85)	3 (1.81)	--	--
	Non-randomized placebo control trial	1 (0.29)	1 (0.38)	--	--	--	--
	Observational	--	--	--	1 (0.60)	--	--
	Single arm study	48 (13.95)	32 (12.30)	5 (14.28)	30 (18.18)	--	--
	Others	40 (11.62)	30 (11.53)	29 (82.85)	130 (78.78)	--	1 (100)
Date of study completion (India)	Applicable only for completed/terminated trials	229 (66.56)	165 (63.46)	33 (94.28)	129 (78.18)	1 (50)	--
	Date missing	19 (5.52)	16 (6.15)	--	7 (4.24)	--	--
	Date mentioned	96 (27.90)	79 (30.38)	02 (5.71)	29 (17.57)	1 (50)	1 (100)
Postgraduate thesis	Yes	12 (3.48)	5 (1.92)	8 (22.85)	24 (14.54)	--	--
	No	332 (96.51)	255 (98.07)	27 (77.14)	139 (84.24)	2 (100)	1 (100)
	Not mentioned	--	--	--	2 (1.21)	--	--
Publication	Published	14 (4.06)	8 (3.07)	2 (5.71)	21 (12.72)	--	--
	Protocol published	--	1 (0.38)	--	--	--	--
	Accepted	1 (0.29)	1 (0.38)	--	--	--	--
	Yes, will publish publication after the study is completed	27 (7.84)	17 (6.53)	--	6 (3.63)	1 (50)	--
	Not yet, not yet decided, not yet published, none yet	54 (15.69)	35 (13.46)	5 (14.28)	4 (2.42)	--	--
	No publication, none, not published, NIL	204 (59.30)	177 (68.07)	26 (74.28)	130 (78.78)	1 (50)	1 (100)
	Not applicable	28 (8.13)	13 (5.00)	2 (5.71)	1 (0.60)	--	--
	Under process, submitted for application, sent to the journal, under publication, underway	6 (1.74)	8 (3.07)	--	--	--	--
	Not mentioned	10 (2.90)	--	--	3 (1.81)	--	--
Brief summary	Present	--	--	35 (100)	165 (100)	2 (100)	1 (100)
	Not mentioned	--	--	--	--	--	--
Individual participant data (IPD) sharing statement	Yes	--	--	1 (2.85)	10 (6.06)	--	--
	No	--	--	27 (77.14)	115 (69.69)	2 (100)	1 (100)
	Not mentioned	--	--	7 (20)	40 (24.24)	--	--

A total of 49 (6%) trials were postgraduate (PG) thesis, two of the trials did not mention, and approximately 93% of trials were not PG thesis. The majority of the trials mentioned publication status as not published or none or not yet published, whereas only 14 trials declared that it is under process or publication, 45 of them declared it as published, two were accepted, and 51 of them showed a positive intent to publish it in the future. Almost all the trials (99.9%) had brief summaries mentioned in the trial registry. Analyzing the section of "Individual Participant Data (IPD) Sharing Statement," it was noted that about 8% of trials agreed to share the IPD statement, 538 trials disagreed, 35 declared it as not applicable, and 169 did not mention anything (Table 4).

The date of study completion (India) was mentioned in only 208 studies and the majority of the studies (n = 557) opted for "Applicable only for Completed/Terminated trials." The days between the registration of the trial in the CTRI and study completion ranged from one day to 739 days with a median (IQR) of 100 (107.25) days.

On analyzing the days between the registration of the trial in the CTRI and the last modification done, it was seen that the time duration ranged from zero to 1166 days, with a median (IQR) of 131 (304). Likewise, the days between the registration of the trial in the CTRI and the first participant enrollment ranged from zero to 1106 days, with a median (IQR) of 7 (6).

Suspended and terminated trials

There were seven terminated trials and five suspended trials registered and almost all of them were interventional in nature. Among the seven terminated trials, three were phase 3 studies, two were phase 2 studies, and two mentioned it as not applicable. The majority of them were randomized studies (n = 6). None of them were PG theses. The interventions used in these trials were molnupiravir, Aviptadil, 8.4% sodium bicarbonate & 5% xylitol, Stempeucel®, alternate nostril breathing & guided meditation, CKD-314, and ursodiol injection. A brief summary of all the terminated trials was mentioned but the reasons for termination were not mentioned.

Among the five suspended trials, three were phase 3, and two mentioned it was not applicable. The majority of them were randomized studies (n = 3). None of them were PG theses. The interventions used in these trials were Kabasura Kudineer, Amukkara Choornam, Nellikai Legiyam, favipiravir, Aceinavir, ruxolitinib, and colloidal silver (NanoAgCide^TM^). A brief summary of all the suspended trials was mentioned but the reasons for suspension were not mentioned.

## Discussion

The present study analyzed the trials registered for research conducted on COVID-19 and coronavirus in the CTRI database. Approximately 807 trials were registered and the majority were interventional studies, followed by observational studies. The majority of the studies were registered in the year 2020, and it showed a decreasing trend till the year 2023. The majority of the studies had a duration under six months. The majority of the studies were related to AYUSH and allied interventions, followed by drug interventional studies and cross-sectional studies. In the drugs used in trials, molnupiravir, colchicine, favipiravir, and ivermectin were a few common molecules used, whereas in biologics and stem cell studies, tocilizumab had the maximum number of trials registered. Among AYUSH interventional therapies, the maximum number of trials were with Kabasura Kudineer, followed by yoga and associated therapies. The majority of the trials mention their phase as not applicable. Analyzing the study designs revealed that the majority of the studies were randomized in nature. There were a very minimal number of suspended and terminated trials in COVID-19 and reasons for suspension or termination were not mentioned in the database.

The duration of the studies was majorly under six months and within that most were registered for four to six months (28%), followed by two to four months (24%), and zero to two months. Another major chunk of the trials was registered with a duration of 10-12 months. The finding was in line with the study conducted by Bhapkar et al. [14]. A study published by Charan et al. reported that about 45% of trials had a duration of under six months; Abinaya et al. and Sathiyarajeswaran et al. too reported similar figures comparable to our study [15-17]. Chaudhari et al. reported that the majority of trials had a duration of three months (43%), followed by three to six months [18]. A study by Bandyopadhyay et al. reported similar figures as compared to the present study, with the majority of trials having a duration of six months (29.1%), followed by three months (28.1%) [19].

Approximately 35% of the registered trials in the CTRI for COVID were for indigenous systems of medicine in India, i.e., AYUSH and associated therapies, and held the majority chunk of the registered studies in the CTRI. An analysis conducted by Raju et al. reported a similar proportion (35%) for the AYUSH studies [20]. An early study by Charan et al. reported a higher proportion (61.5%) as compared to the present analysis [16]. Similar higher figures (55.3%) for AYUSH-related trials were also reported by Bandyopadhyay et al. [19]. Rao et al. mentioned that 67 out of 122 studied trials were from the traditional system of medicine [21]. However, these studies were conducted in the very early stage of the pandemic in the year 2020. Chaudhari et al. mentioned a similar higher proportion (49%) for the interventional trials related to AYUSH therapies, and it is noteworthy to mention that about 42% were funded by the Ministry of AYUSH [18]. Similar data were also reported by analysis done by Sathiyarajeswaran et al. [17]. A study conducted by Bhave et al. reported a lower proportion (26.53%) of studies registered for AYUSH-related interventions as compared to the present analysis [22].

The heightened level of registration for AYUSH and associated studies suggests that for a country like India, the therapists and researchers primarily opted to utilize its indigenous systems of medicine during the pandemic. Moreover, there was a major thrust from the Ministry of AYUSH, Government of India to conduct research and also collaborate with other national research bodies, national and state institutes, and also with the pharmaceutical industry. To pursue the same, the Ministry of AYUSH set up an Interdisciplinary AYUSH Research and Development Task Force to collaborate and coordinate to develop potential effective preventive or curative therapy to counter COVID-19 [23]. To follow on the same, a press release by the Government of India, AYUSH mentioned that “Across numerous research organizations and national institutes under the Ministry of AYUSH, there are ongoing research endeavors at 141 centers nationwide, encompassing 120 studies focused on AYUSH interventions as preventive measures,” dated 23 March 2021 [24].

Given that the disease was novel and there were no pharmaceuticals available from conventional medicine for addressing this condition, it was taken as a crucial opportunity to develop drugs derived from the indigenous system of Indian medicine and showcase their efficacy in the management of COVID-19.

In the analysis of interventions used under the AYUSH and associated therapies, the majority were registered for Kabasura Kudineer combination therapies, yoga and allied therapies, AYUSH-64 combination therapies, homeopathic therapies, chyawanprash, and ashwagandha. In these, about 176 trials were related to Ayurvedic preparation and were the most commonly used therapies among the indigenous medicines. Charan et al. reported that almost 70% of the trials were of Ayurvedic preparations, followed by homeopathy and Siddha. They also reported that among them, the interventions most commonly investigated in the trials included Arsenicum album, ashwagandha, AYUSH-64, and Guduchi Ghan Vati [16]. Bhapkar et al. in a study reported that Guduchi Ghana, Sanshamani Vati, Amritadi Guggulu, ashwagandha, chyawanprash, Anu Taila, AYUSH Kwath, Yashtimadhu, and AYUSH-64 were common interventions used for COVID-19 in the studies registered in the CTRI [14]. A similar study reported Guduchi, ashwagandha, Yashtimadhu, AYUSH-64, AYUSH Kwath, curcumin, and chyawanprash as the common interventions used for COVID [25]. Raju et al. reported a lower proportion (25%) of trials of COVID-19 registered with Ayurveda and yoga therapies [20].

In the present analysis, the drug interventional trials accounted for 15%, and frequently used molecules were molnupiravir, colchicine, favipiravir, ivermectin, dexamethasone, and immunocin α. Whereas, biologics and stem cell therapies accounted for 3.4% of the trials registered and common molecules on trial were tocilizumab, convalescent plasma, anti-SARS-CoV-2 hyperimmune intravenous immunoglobulin, baricitinib, ruxolitinib, tofacitinib, Stempeucel®, etc. Bhave et al. reported that approximately 40% of the trials were on modern medicine and it included drugs, biologics, stem cell therapies, vaccines, etc. [22]. Charan et al. also reported that about 31% of trials were from allopathic interventions [16]. Chaudhari et al. reported that about 1/5th of the studies in the CTRI were from allopathy and these figures were comparable to our study [18].

The crucial point that needs to be highlighted is the interventions used for the prevention or therapy of COVID-19 were mostly repurposed drugs or other complementary and alternative medications or therapies. The use of novel molecules was comparatively lesser and that too the number of repurposed molecules in allopathy or modern medicines was lower when compared to AYUSH therapies.

In the present analysis, the majority of the studies registered declared their trial phase as "N/A," followed by phase 2 and phase 3 trials. Raju et al. reported a similar finding with almost more than half of the trials mentioning not applicable, followed by phase 2 (17%) and phase 3 (12.5%) [20]. A similar study reported that almost 24% of trials were therapeutic exploratory and 16% were therapeutic confirmatory trials [17]. Our study finding was also supported by the reports of Charan et al. and Chaudhari et al. whereas Bhave et al. reported phase 3 trials to be more common than registered phase 2 studies [16,18,22].

In this analysis, the majority of the registered studies were randomized studies (54%), and among them, randomized parallel-group trials were the most common. Abinaya et al. reported that a major chunk (64.16%) of interventional trials was randomized [15]. A similar study by Sathiyarajeswaran et al. reported that about 38% of the COVID-19 trials were randomized [17]. Raju et al. reported that about 17.30% of the studies were randomized parallel-group trials [20]. The greater number of randomized studies registered for COVID-19 is a crucial finding as it denotes proper designing of trials for the generation of evidence as randomized trials can generate higher quality of evidence as compared to the non-randomized ones.

In comparison to the data collected from the CTRI, a study conducted on the collected data from the published literature and ClinicalTrials.gov database reveals that the majority of the studies were conducted on synthetic drugs unlike in our case [26]. In the same study of the published randomized controlled trials (RCTs), herbal medicines have been the subject of 23 trials (10.6%) and 357 registered RCTs (12.7%). It was also mentioned that of the 78.7% of registered studies on herbal medicines, the majority of the research is concentrated in India (31.9%), China (17.1%), and Iran (106, 29.7%), and the commonly used herbal medications in registered randomized trials were curcumin, Nigella sativa, licorice, ginger, AYUSH-64, and artemisinin. As observed in our study, the commonest trial phases in other RCTs and registered trials across various regions of the world were phase 2 (29.3%) and phase 3 (24%), whereas about 10% of the trials mentioned it as not applicable [26]. Similar data were also presented by a study conducted on the CT.gov database mentioning about 43.4% of the total registered studies were in phase 2 or phase 3 [27]. In the same study, it was reported that the medications that have been tested most frequently in various clinical trials were plasma, hydroxychloroquine, azithromycin, tocilizumab, ivermectin, favipiravir, remdesivir, ritonavir, lopinavir, plasma, and interferon [27].

The majority of the registered trials disagreed to share their patient data. A brief summary of the study was provided by the majority of trials but there was no update of the trial progress or last status of the registered trial. The date of study completion was mentioned in only about 1/4th of the registered trials. Similarly, the last modification timeline from the date of registration was also not updated to the completion of the study. The median days between the registration of the trial and study completion were 100 days and the median days between the registration of the trial and the last modification done were 131 days. Both the time duration shows more than three months available for the registered studies to update their information in the CTRI database but the majority of them did not do so. Sharing data or updating the database would benefit other researchers to keep track of the current evidence before planning their research. These small changes can help improve the completeness of the information shared in the database.

On assessing the database for the suspended and terminated trials for COVID-19, the present analysis could find very few studies in this category, which is a crucial finding. A similar finding was also observed from the study conducted on a larger database, which reported that only 0.8% and 1% of the trials were suspended and terminated, respectively [26]. Likewise, a study by He et al. on CT.gov reported low rates of suspended (0.69%) and terminated (0.90%) trials [27]. A trial becomes successful if it has proper planning, appropriate designing, robust methodology, adequate feasibility, and proper conduction following the protocol and guidelines. Here, a minimum number of terminated or suspended trials means the successful conduction of the trials, which is a positive finding. However, the reasons for the termination or suspension of those trials were not mentioned. Mentioning the reasons for the termination or suspension of any trial would help warn the researcher to plan and check the feasibility of their study before starting their project and would help to save a huge amount of financial and other resources.

Strength and limitations

This study has tried to comprehensively review all the existent information present in the CTRI database and summarize them. It has included all the data till September 2023 and has identified the suspended and terminated trials related to COVID-19. This concise analysis would help to extract information regarding the conducted trials and identify interventions and methods to plan research studies in the future. There are a few limitations, like we relied on the trial search function of the database with predetermined keywords for extraction of data, and this could have limited the search because of an error in indexing in the database. Secondly, in many sections of the analysis, some of the data was missing. However, we expect these errors to be very minimal and that would not affect the outcomes of the study. We have analyzed the completeness of the information provided in the CTRI database and the methodological qualities and outcomes of these registered trials could not be assessed and were out of the scope of this article as these data were not mentioned in the database.

Declaration

The data presented in this study are solely based on the data mentioned and uploaded by the investigators in the CTRI database. The data provided here do not represent the opinion of the CTRI.

## Conclusions

The current study has tried to effectively summarize the trials registered in the CTRI database that were conducted in India. The information generated could help the researchers, institutions, governing authorities, and others to get a concise overview of the registered trials and interventions used for prophylaxis or management of COVID-19 and can help in planning and conducting future research in any condition with similar pathophysiology. The noticeable finding from the analysis was the predominant use of therapies of Indian traditional medicines as compared to modern medicine in the trials for COVID-19 and this was because there were no effective therapies available even in modern medicine for this unforeseen menacing disease condition. This is a crucial finding and has set an example for the use of various Indian traditional medicines in various disorders as a result of enhanced thrust from the authorities to conduct research with AYUSH therapies. The quality of the studies could not be assessed and the study findings were restricted to the information provided in the database. The majority of the registered studies were randomized in nature and the proportion of suspended and terminated trials was very low, denoting proper conduction and designing of the trials leading to proper usage of invaluable resources.

## Appendices

**Table 5:** Summary of AYUSH interventions used by studies registered in the CTRI for COVID-19 AYUSH: Ayurveda, Yoga and Naturopathy, Unani, Siddha, and Homeopathy along with other allied interventions in these categories; CTRI: Clinical Trials Registry - India; ICMR: Indian Council of Medical Research; RNB: Raj Nirvan Bati; SOC: standard of care.

S. No.	AYUSH intervention	Number of trials
1	Combination of Dashamula Kwatha and Pathyadi Kwatha with Trikatu Churna, Sansamani Vati, AYUSH-64, and Yastimadhu Ghanavati	2
2	Combination of Adathodai Kudineer, Thalisathi Chooranam, Thulasi Chooranam, Pavala Parpam, Brahmananda Bairavam, Mathirai, and Thoothuvalai nei	2
3	Combination of Arsenic Album 30c potency, Bryonia alba 30c potency, Camphora 1M potency, coronavirus-related nosodes (30c potency), and matching placebo pills size 30 homeopathy medicine	1
4	Combination of Bilwadi Yog, Kantakaryavaleha, and Shadangodak	2
5	Habbe Zehar Mohra and Habbe Tabasheer	2
6	Immunofree 500 mg tablets and Reginmune 750 mg capsule	2
7	Infuza and Kulzam	2
8	Aceinavir (1-gram uncoated tablet)	2
9	Alternate nostril breathing & guided meditation	2
10	Amrutha Sanjeevini tablets plus Ojovardhini capsule (test formulation B)	1
	Jeevaneeyam tablets plus Ojovardhini Capsule (test formulation A)	
11	Amukkura Chooranam tablet each tablet 500 mgs and Nellikai legiyam	2
12	Ayurvedic regimen (details not mentioned)	4
	Ayurveda formulation (details not mentioned)	
13	Anu Taila	2
14	Aragwadhadi Kwath mentioned in bhavprakash jwarachikitsa	1
15	Arathai Kudineer standard of care	1
	Notchi Kudineer standard of care	
16	Arogya Kashayam-20	1
17	Arsenic Album 30 or Arsenic Album 200 add-on to standard care	4
	Arsenicum album 30 Influenzinum 30 Oscillococcinum® (Anas barbariae hepatis et cordis extractum 200K)	
	Arsenicum album 30, Oscillococcinum® (Anas barbariae hepatis et cordis extractum 200K), Influenzinum 30)	
	Arsenicum album 30c	
18	As standard treatment suggested by WHO/ICMR (treatment period-14 days)	4
19	Ashwagandha	5
	Ashwagandha tablet and Shunti capsule	
20	Ayur Raksha kit	2
21	Ayurveda decoctions and Panchagavya	2
22	Ayurveda intervention (Bharangyadi kashayam, Sudharshanavati, Guduchi paaneeyam, Thaleesadi choornam for 21 days)	2
23	Ayurveda therapy consisting of Pranvir capsule and capsule Yashada Rasayana	2
24	Ayurvedic drug RNB	1
25	Ayurvedic polyherbal formulation	2
26	Ayurvedic Suved, Reimmugen, colostrum	2
27	Ayush-64	11
	Ayush-64 capsule, Sanshamani Vati, Vatashleshma, Jwarhar Kwatha	
	Ayush-64, Agasthya Haritaki, Anu Taila nasal drops, and Broncho-T granules	
	AYUSH-64, Yashtimadhu, and Sanshamani Vati	
28	Ayush Intervention - Treatment Protocol - I	2
	Ayush Intervention - Treatment Protocol - II	
	Ayush Intervention - Treatment Protocol - III	
	Ayush Intervention - Treatment Protocol - IV	
	Ayush Intervention - Treatment Protocol - V	
	Ayush Intervention - Treatment Protocol – VI	
29	Ayush medicine NOQ 19	3
30	Bryonia alba 30C	1
31	Camphora 1000 C	4
32	ASMO	2
33	Immurich	2
34	AEV01 100 mg	2
35	Chyawanprash	7
	Dabur Chyawanprash, Giloy ki Ghanvati, Kalmegh Tablets, and Tulsi tablets	
	Standard Preventive Regimen plus Ayurveda Rasayana (Chyawanprash)	
36	Clevira	2
37	Malla Chandrodaya & Ashthadashang Ghana vati with anupana of Parijata Swaras	1
38	Dashanga gulika & Haridra Khanda	2
39	Deep breathing Pranayama breathing technique and conventional allopathic treatment	1
40	Drug interventions (Ayush Kwath)	4
41	ES16001	2
42	Formulation 2 and SOC	3
	Formulation 3 and SOC	
43	Gayatri Mantra chanting and Pranayama along with the usual treatment	2
44	Ayurveda as an add-on to standard care	2
45	Guduchi Ghan Vati	6
46	Homeopathic medicine	9
	Homeopathy medicines, Arsenicum album, Bryonia alba, Rhus Toxicodendron, Belladonna Gelsemium, and Eupatorium Perfoliatum	
	Homeopathic medicine: Arsenic Album, Bryonia Alba, Gelsemium, Antimonium Tartaricum, and Crotalus Horridus	
47	Immunity Kit	3
48	Immurise	2
49	Indicated homeopathic medicine in centesimal potency	2
50	Jeevaneeyam tablets plus Ojovardhini capsule (Test Formulation A), Amrutha Sanjeevini tablets plus Ojovardhini capsule (Test Formulation B)	1
51	Joshanda (Decoction)	1
52	Kabasura Kudineer and Nilavembu Kuidneer	23
	Kabasura kudineer	
	Bramanandha bairavam, Adathodai Manapagu, Amukkara Chooranam, Kapasura Kudineer, and Thalisathy Vadagam	
	Kabasura Kudineer, Tab. Amukkarachooranam, Tab. Athimathuram, Tab. Brahmanandha Bairavam, Adathodai Manappagu, Thippili Rasayanam, Notchi Kudineer	
	Kabasura Kudineer, Nilavembu Kudineer, Amukra Churnam, Thalisadhichurnam, Adathodai Manappagu, Brahmanada Bhairavam Pills, Thippili Rasayanam, Maldevi Chenduram, Adathodai, and Kudineer Nochi	
	Kudineer, Thirikadugu Churnam, Adathodai Manappagu, and herbal tea	
	Kabasura Kudineer Chooranam, Amukkara Chooranam Mathirai, Thaleesadhi vadagam, Adathodaimanapagu and Brammanandha bairavam	
	Kapasura Kudineer, Amukkara Chooranam tablet, Thalisathy Vadagam, Bramanandha Bairavam tablet, and Adathodai Manapagu	
53	Khameera Marvareed, Unani Joshanda/Decoction	2
54	Ledum palustre 200CH	1
55	Ayurvedic nasal spray	1
56	Maha-Sudarshana Ghan Vati	1
57	Medihope	2
58	Meditation and breathing exercises	2
59	Mind-body techniques	4
60	MSL	2
61	Mulmina mango drink plus standard-of-care treatment	2
62	MV Kashayam	4
63	Nasoshield nasal spray, Tulsi and Mulethi extract	1
64	Neutral immersion bath	1
65	NF 2 Ayurvedic propreitary medicine	2
66	Notchi Kudineer standard of care and Arathai Kudineer standard of care	1
67	Panchamutti Kanji	2
68	Piper betle with the combination of Swarnabhasma (Herbo mineral combination)	2
69	Pippali Rasayana, Nagradi Kashaya Ghana Vati, and ICMR-directed therapy	2
70	A pragmatic clinical study that studies the whole system personalized approach of medicine practice	2
71	Pranayama and Yoga Nidra in addition to routine activities	2
72	Raj Nirwan Bati (RNB) capsule	3
73	Samshamani Vati, Anutaila, Ayush Kadha	4
	Shanshamani Vati or Sudarshana Ghanavati or Ashwagandha	
74	ShatPlus along with standard treatment	1
75	Shirashadi Kasai	1
76	Single arm, uncontrolled, pretest post-test efficacy design	3
	Standard care and Yoga intervention	
77	Standard care for moderate and severe COVID-19 subjects plus Tab. Gorochanadi Vati 125 mg	2
78	Standard drug regimen	2
79	Standard of care plus Haldi 30 drops	2
80	Study inhaler	2
81	Sudarshan Kriya	2
82	Swarna Amalaki as an adjuvant therapy	2
83	Swarnavir Fifatrol	1
84	Swas Vimochan, SwasanRakshak, Swasamrite, and immune energy tablets	2
85	Pinak	1
86	Bresol and Septilin	1
87	CoReach	2
88	Immusante and Guduchi	2
89	Bhoumya and Saathmya	1
90	Tadios along with standard-of-care treatment	2
91	Thuja occidentalis 200 CH	1
92	Tulsi tablets, Giloy ki Ghanvati, Dabur Chyawanprash, and Kalmegh tablets	1
93	Turmgel Mouth Dissolving Lozenge 100 mg	1
94	Unani herbal formulation in tablet form	2
95	Under behavioral therapy, under the food additives category, and under physical therapy	2
96	Vasa Ghana (aqueous extract of Adhatoda vasica), Guduchi Ghana (aqueous extract of Tinospora cordifolia), Vasa Guduchi Ghana (whole water-soluble extract): 500 mg (250 mg each of Vasa and Guduchi)	1
97	Viranorm plus standard of care (SOC)	2
98	Virunil 500 mg capsules	1
99	Vyaghradi Kashay tablet	2
100	Vyaghri Haritaki	1
101	Yoga-based rehabilitation including loosening exercises, breathing exercises, asanas, pranayama, and guided relaxation	15
	Yoga modules for management of coronavirus disease 2019 (self-as-control)	
	Yoga with standard care	
	Yoga therapy	
	Online yoga intervention, yoga cum herbal concoction	
	Tele yoga	
102	Zincum Muriaticum 200C	2
103	ZingiVir H	3
104	Therapy not mentioned	22
